# Barriers and facilitators to providing primary care-based weight management services in a patient centered medical home for Veterans: a qualitative study

**DOI:** 10.1186/s12875-015-0383-x

**Published:** 2015-11-14

**Authors:** Melanie Jay, Sumana Chintapalli, Allison Squires, Katrina F. Mateo, Scott E. Sherman, Adina L. Kalet

**Affiliations:** VA NY Harbor Healthcare System, 423 East 23rd Street, New York, NY 10010 USA; NYU School of Medicine, 550 1st Avenue, New York, NY 10016 USA; NYU College of Nursing, New York, NY 10003 USA

**Keywords:** Obesity, Veterans, Weight management, Primary care, Key informant interviews

## Abstract

**Background:**

Obesity is highly prevalent among Veterans. In the United States, the Veterans Health Administration (VHA) offers a comprehensive weight management program called MOVE!. Yet, fewer than 10 % of eligible patients ever attend one MOVE! visit. The VHA has a patient-centered medical home (PCMH) model of primary care (PC) called Patient-Aligned Care Teams (PACT) at all Veterans Affairs (VA) Medical Centers. PACT teamlets conduct obesity screening, weight management counseling, and refer to MOVE!. As part of a needs assessment to improve delivery of weight management services, the purpose of this study was to assess PACT teamlet and MOVE! staff: 1) current attitudes and perceptions regarding obesity care; 2) obesity-related counseling practices 3) experiences with the MOVE! program; and 4) targets for interventions to improve implementation of obesity care in the PC setting.

**Methods:**

We recruited 25 PACT teamlet members from a single VA study site—11 PC physicians, 5 registered nurses, 5 licensed practical nurses, 1 clerical assistant, and 3 MOVE! staff (2 dietitians, 1 psychologist)—for individual interviews using a combination of convenience and snowball sampling. Audio recorded interviews were professionally transcribed and iteratively coded by two independent reviewers. The analytic process was guided by discourse analysis in order to discover how the participants perceived and provided weight management care and what specific attitudes affected their practices, all as bounded within the organization.

**Results:**

Emerging themes included: 1) role perceptions, 2) anticipated outcomes of weight management counseling and programs, and 3) communication and information dissemination. Perceived role among PCPs was influenced by training, whereas personal experience with their own weight management impacted role perception among LPNs/RNs. Attitudes about whether or not they could impact patients’ weight outcomes via counseling or referral to MOVE! varied. System-level communication about VHA priorities through electronic health records and time allocation influenced teams to prioritize referral to MOVE! over weight management counseling.

**Conclusion:**

We found a diversity of attitudes, and practices within PACT, and identified factors that can enhance the MOVE! program and inform interventions to improve weight management within primary care. Although findings are site-specific, many are supported in the literature and applicable to other VA and non-VA sites with PCMH models of care.

## Background

Approximately 35 % of adults in the United States have a Body Mass Index (BMI) in the obese range [1] putting them at risk for obesity-related comorbidities that are usually treated within primary care (PC) settings. The PC setting is an important venue to promote weight management and other healthy lifestyle behaviors. Research demonstrates that counseling by primary care providers (PCPs) improves weight management behaviors [2]. The United States Preventive Services Task Force (USPSTF) recommends screening for obesity and intensive counseling, but primary care providers often fail to counsel patients about weight management for many reasons including constraints on time [3] and lack of training [4, 5]. Further, many PC settings do not offer intensive weight management programs.

The Veterans Health Administration (VHA), the only singer-payer health care system in the United States, has been a leader in implementing systematic BMI screening and evidence-based weight management services. In 2006, the VHA implemented a comprehensive weight management program called MOVE! at all VHAs nationwide [6, 7] to address the high rates of obesity in the Veteran population [8, 9]. As part of this program, all patients are supposed to be screened for obesity within primary care (94 % screening rate) [10] and if necessary, offered behaviorally focused weight management treatment via separate MOVE! visits. MOVE! is the largest weight management program offered by an integrated healthcare system, and over 132,000 patients attended MOVE! visits in 2013 [10]. A MOVE! visit usually involves individual- or group-based self-management programs and/or a telephone-based adaptation called TeleMOVE!. A recent study at the Los Angeles VA showed that MOVE! participants who went to 3 or more MOVE! sessions lost an average of 2.2 kg at 1 year, despite having gained an average of 1.4 kg the year prior to enrollment [10]. However, only 8 % of eligible patients ever attend a single MOVE! session, suggesting significant underutilization of the program [11, 12]. Reasons for this underutilization are unclear but allude to implementation problems in the primary care setting – an integral referral point for the program. Indeed, 90 % of all VHA patients are seen in primary care with an average of 3.6 visits per year [13], providing multiple opportunities for weight management counseling and referral to MOVE!. The UK National Health Service offers various lifestyle-based weight management programs including a primary care-based program called Counterweight as well as commercial programs [14, 15]. These programs appear to have similar challenges and outcomes as the MOVE! program. For instance, a randomized trial comparing 8 UK-based weight management interventions for patients recruited through their general practitioner found that only 8.3 % agreed to enroll in the study. A completers only analysis of the programs showed that the mean weight loss varied between 1.2 and 4.4 kg at 1 year [16].

To improve counseling opportunities and enhance care coordination during a primary care visit, the VHA adopted a patient centered medical home (PCMH) model called Patient Aligned Care Teams (PACT) in 2010 at all Veterans Affairs (VA) Medical Centers. According to the National Center for Quality and Assurance, more than 10 % of U.S primary care practices are recognized as PCMH sites [17]. The PCMH model, designed to provide care that is team-based and more patient centered [18], potentially expands the number of personnel that can work together to improve obesity-related patient care [19] without relying solely on the PCP. At the VA, the PACT “teamlet” consists of 4 members—a clerical assistant, a primary care provider (PCP), a registered nurse (RN) care manager (usually has associate or bachelor-level professional nursing degree), and a licensed practical nurse (LPN—usually has a 1-year practical nursing degree). However, no studies that we know of have examined current weight management and lifestyle counseling-related attitudes and practices by PACT teamlets. In fact, a recent editorial commented that there were few, if any, studies focused on how the PACT model can promote patient-centered interactions around healthy behavior change [20].

The recent VHA implementation of PACT at all VA sites provides the opportunity for long-term team-based care that is integrated with the MOVE! program. However, in the 2013 MOVE! progress report, the majority (55 %) of VA sites surveyed reported that MOVE! programs were separate from PACT [10]. Improving weight management counseling by PACT teamlets could increase patient motivation to attend the MOVE! program and facilitate weight loss for those who do not attend. Furthermore, since both PACT teamlets and MOVE! staff are encouraged to use goal setting to promote behavior change, efforts to increase adoption of this technique may promote synergy of obesity care between MOVE! and PACT. Goal setting is a theory-based method [21, 22], that has been successfully used to promote behavior change [23]. We have previously found in focus groups with Veterans that goal setting-based interventions may facilitate lifestyle behavior change in primary care [24]. Current provider attitudes toward and practices around goal setting for patients at VA sites, however, are unclear [25].

Previous MOVE! studies have not sufficiently explored how individuals working in primary care affect attendance and adherence to the program [26–29]. A recent study surveyed 745 primary care and MOVE! staff about their perceptions on Veterans’ attrition from the MOVE! program, but did not explore how the staffs’ obesity-related attitudes and counseling practices may impact attrition [29]. Similarly, two other studies looked at interviews with MOVE! coordinators, but did not explore attitudes and practices of primary care staff [26, 28]. In contrast, a study that did interview primary care providers in addition to MOVE! staff and leaders [30] found that staff believed obesity care to be important, but some doubted the effectiveness of MOVE!. Notably however, this study was conducted prior to PACT implementation and thus did not explore specific counseling practices by primary care teams. Our study addresses these gaps in the literature and was conducted as part of a needs assessment for future intervention development to improve lifestyle-focused weight management services within the PCMH model of care at the VA.

## Methods

We chose a qualitative approach for this study in order to elicit the full range of staff experiences so we could better understand how the PCMH model can improve obesity care and adherence to the MOVE! program. The purpose of the study was to assess PACT team and MOVE! staffs’: 1) current attitudes and perceptions regarding obesity care; 2) obesity-related counseling practices, in particular the use of goal setting; 3) perceptions and experiences with the MOVE! program; and 4) potential targets for interventions to improve obesity care in the PC setting at the VA.

The focus of the study was to examine how members of PACT teamlets and MOVE! staff communicate their weight management counseling experiences, how they both perceive and provide weight management care at the VA, and what specific attitudes affect their care practices, all as bounded within the organization. We also aimed to explore differences between and among the various professions. Organizational studies research has long recognized that the organization where care takes place influences healthcare providers’ and their experiences. Thus, a discourse analysis approach was adopted to guide the analytic process. In discourse analysis, researchers analyze the participants’ use of language in relation to their interactions within an organization [31, 32]. Given the complexity of weight management in the primary care context, the approach is well suited when attempting to understand institutional norms, role expectations, and ideals of individuals working in the PC setting at the VA. All research procedures were approved by the VA New York Harbor Healthcare System (NYHHS) Manhattan campus Institutional Review Board.

### Sample selection

Based on prior studies [26, 27] and consistent with the study design, we anticipated that we would need between 20 and 25 participants but planned to recruit more if data saturation was not achieved. At the Manhattan VA, serving as our single study site, one to four PCPs may work with the same RN and LPN. For this reason, we anticipated that more PCPs would be recruited than nursing staff. We focused on obtaining the perspective of nurses (LPN and RN), primary care providers (MD and NP), and MOVE! staff because they are directly involved with screening and treatment of obesity.

Purposive sampling [33] was used to select participants based on their professional role within a PACT team/teamlet or their leadership role at the VA study site. Subsequent snowball and convenience sampling [33] further allowed us to expand the group of participants included in the study. Participants were identified through key stakeholders and by asking other participants to suggest colleagues who may have valuable insight and/or leadership positions within the organization. Participants were approached in person by the Principal Investigator (MJ) at staff meetings and research staff then sent follow up emails and made phone calls to confirm recruitment and schedule visits. Since the PI also cares for patients as an MD within PACT, staff working directly with her were excluded from the study.

### Interview procedures

With the consultation of an outside qualitative methods expert with a human resources for health research background (APS), we developed and piloted a semi-structured interview guide that included open-ended questions to explore the 4 domains listed in Table 1. We used open-ended questions related to participant attitudes and experiences with obesity counseling, goal setting, and the MOVE! program. Questions aimed to elicit discussions of perceived barriers to patient weight loss, the perceived role of staff in weight management, and provision of weight management counseling. Some items were similar to those used in a VA patient study [25]. We also collected basic demographic, professional, and training data.

**Table 1:** Interview guide domains and example questions/probes

Domain	Sample questions/probes
Perceptions/experiences with MOVE! Program	• What aspects of the MOVE! program are most effective? (Probes: referral process, curriculum, patient follow up, tell me about a patient who was very successful through the MOVE! program)
Perceptions about role of PACT members or how PACT teams care for patients	• What is the current role of the PACT teamlet (PCP, RN, LPN,) in helping MOVE! patients to lose weight? • How could the VHA better facilitate the PACT teamlets in helping patients to lose weight?
Perceptions about role of PACT members in weight management	• How can we improve weight management at the VA? (Probes: VA’s role in practitioner facilitation, given all of your other responsibilities, what do you think your role should be in helping patients lose weight?)
Barriers, facilitators, and practices pertaining to goal setting with patients	• How useful is goal-setting as a tool for behavior change? • What are some barriers to using goal-setting to promote behavior change in Veterans? • How can we better facilitate goal setting from providers at the VA?

*Abbreviations*: *PACT* patient-aligned care teams, *MD* primary care doctor, *NP* nurse practitioner, *RN* registered nurse, *LPN* licensed practical nurse, *MOVE!* MOVE! program staff, *VA* Veterans Affairs Medical Centers

We conducted individual interviews in a private office convenient to but not in the participants’ work location. For each session, written informed consent was obtained to participate in an audio-recorded interview. Participants were assured that their participation was voluntary and that efforts would be made to ensure that their information was kept confidential. Each interview lasted approximately 45–60 min and was led by the PI with an additional team member present to take extensive field notes. Participants were compensated for their time with a $75 professional development credit to be used toward patient-care enhancements, such as books or clinical tools. All interviews were conducted between June and October of 2013.

Audio recordings of each session were professionally transcribed. Research staff then removed all identifying content and corrected mistakes in the interview transcripts.

### Data analysis

Alvesson and Karreman’s formulation of a *close-range/determination* approach structured the discourse analysis [34]. This approach carries an assumption that discourse can illuminate local construction of feelings, norms, and ideals of a given topic within a system and thereby enabled us to discover how participants discussed their attitudes and practices around lifestyle and weight management counseling with Veterans [34, 35]. We first developed an initial codebook based on field notes, and two coders (MJ & SC) separately reviewed and coded initial transcripts using NVivo 8 software [36]. The coders modified the codebook as new codes emerged, met frequently to compare disagreements, and negotiated final codes to achieve consensus that resulted in themes and categories. Research team discussions, transcript highlights, and field notes were used to help synthesize themes based on coded content. APS provided objective review of the work, since she did not participate in data collection, and facilitated the consensus process. The process occurred over a period of several months. Representative quotes from the transcripts were chosen to illustrate various the themes and categories that emerged from the analysis. A summary of the results of the study were emailed to the participants to elicit thoughts on findings and ensure that participants would be aware of the results.

## Results

Research staff contacted 34 potential participants via email or phone, and 9 (4RNs, 2LPNs, 2MDs, and 1 Other) either declined or did not respond. Common reasons for declining included not wanting to be audio-recorded and not having sufficient time to participate. The final sample that achieved data saturation comprised 25 interviews with 11 PCPs (MDs or NPs), 5 RNs, 3 MOVE! staff (RDs and behavioral psychologist), 5 LPNs, and 1 clerical assistant. Six participants held an additional managerial role. Participants came from 11 of 13 PACT teams (not including resident PACT teams) at the site. Table 2 shows additional demographic information. Feedback from the participants about the results was generally positive, and no major concerns were raised.

**Table 2:** Demographic information of participants

	MD/NP *n* = 11	RN *n* = 5	LPN *n* = 5	MOVE! *n* = 3	Total^a^ *n* = 24
Age (years)					
Range	40–51	36–63	27–62	28–43	27–63
Average	45.9	47.4	44.4	36.7	45.0
Gender	*n* (%)	*n* (%)	*n* (%)	*n* (%)	*n* (%)
Male	3 (27)	1 (20)	1 (20)	0 (0)	5 (21)
Female	8 (73)	4 (80)	4 (80)	3 (100)	19 (79)
Race					
White	8 (73)	2 (40)	0	3 (100)	13 (54)
Black	0 (0)	2 (40)	5 (100)	0 (0)	7 (29)
Asian	3 (27)	1 (20)	0	0 (0)	4 (17)
Ethnicity					
Hispanic/Latino	0 (0)	2 (20)	0 (0)	0 (0)	1 (4)
Non-Hispanic/Latino	11 (100)	3 (80)	5 (100)	3 (100)	23 (96)
Average years in NY HHS	10.1	12.4	8.8	5.2	10.3
Average years in any VA	11.3	15.0	9.0	5.2	11.3
Average years practicing since licensing	17.0	19.8	14.2	12.3	16.7

Age, gender, race, ethnicity, average working years at the VA NY Harbor Healthcare System and any Veterans Health Administration, and average years practicing since licensing. Participants organized by license: MD = primary care doctor, NP = nurse practitioner, RN = registered nurse, LPN = licensed practical nurse, MOVE! = MOVE! staff (2 registered dieticians and 1 psychologist)

^**a**^Total excludes data from 1 interviewed clerical assistant to protect identity

Below, we report our findings starting with the description of current weight management practices described by participants, followed by a description of themes that emerged during the discourse analysis that explained current weight management attitudes and practices. We also review barriers and facilitators to providing obesity-related care that emerged from the interviews. We synthesized the three major themes as: 1) Role perceptions, 2) Anticipated outcomes of weight management counseling and programs, and 3) Communication and information dissemination. We found that each theme could be further categorized into Local/National VHA *System-Related Factors* and *Individual/Team Factors*.

### Weight management-related processes and practices by PACT teamlet members and MOVE! staff

PACT teamlets each described processes through which they delivered weight management-related care. On arrival, the patient first encountered the clerical assistant who checked in the patient. Then, the patient saw the nurse (RN or LPN) for 5–20 min in order to take vital signs including weight and height. If the BMI was ≥25 kg/m^2^ with co-morbidities, or ≥30 kg/m^2^, the electronic medical record (EMR) generated a reminder for the staff person to discuss the risks of excess weight and offer the MOVE! program. The patient then saw the PCP for 40 min (if a new patient) or 20 min (if a revisit). Either the nurse or the PCP could complete the tasks to close the EMR reminder. There was a great deal of variability in how and whether nurses or PCPs gave specific lifestyle and weight management advice. In some cases, the PCP counseled the patient about lifestyle and weight, promoted the MOVE! program, and/or referred the patient to an individual appointment with a dietitian. The clerical assistant would then typically see the patient after the visit to schedule/confirm upcoming appointments. This person also provided flyers and handouts about the MOVE! program detailing where to go and when. Dietitians or psychologists specializing in behavioral weight loss saw patients either during group visits as part of the MOVE! program and/or individually.

#### Goal setting

PCPs, RNs, and MOVE! staff discussed how they received training about the importance of SMART (small, measureable, achievable, realistic, and timely) goals [37] and motivational interviewing techniques to address patient barriers to achieving health goals. Despite these trainings, the majority of nurses and doctors did not report using goal setting strategies regularly with patients. On the other hand, the MOVE! staff all reported frequently using goal setting strategies with patients.

### Theme 1—role perceptions

#### System level factors

A facilitator of obesity care was that all of the RNs and PCPs had received training in motivational interviewing and goal setting as part of a national VHA initiative to improve behavior change counseling. Two of the RNs had then trained 2 of the LPNs in these techniques as part of a previous pilot project to increase lifestyle counseling and MOVE! attendance (this project had ended several months prior due to staffing changes as a result of damage to the facility by Hurricane Sandy). However, there were several barriers to using these techniques in a clinical setting to facilitate weight management in patients. The majority of RNs and LPNs expressed frustration that they often did not have sufficient time to provide lifestyle and weight management counseling. Patients often did not realize that they were supposed to come in 20 min before their PCP appointment so that they could meet with the nurses, resulting in rushed nursing visits. LPNs and RNs also cited staffing issues, sometimes having to cover more than 1 teamlet. For PCPs, they only had limited time to address many competing patient issues. Despite this, a few RNs and MDs described how they were able to fit in counseling when they had extra time in their schedule due to a cancellation or a visit scheduled specifically for weight management counseling. Perceptions about what their license allowed them to do influenced others, especially the LPNs. One of the LPNs said that she did not counsel patients because it involved assessing current behaviors, “I do most everything except anything involving assessment, like preventative health assessment. The LPN is --we’re not allowed to assess” (LPN 2).

#### Individual/Team level factors

A major facilitator to weight management counseling by PACT teamlets was having the belief that counseling patients about weight was part of their role and responsibility. For PCPs, perceived competency influenced this role perception; having the belief that they had received sufficient training in medical school and residency influenced perceived role,“ I mean having some training, just even having something to fall back on, the plate method and the 24 h recall, it’s just something that I feel comfortable being able to do” (MD 5). Some of the doctors did not feel that they should provide counseling due to lack of nutrition education “I just don’t think we’re equipped to do that. I don’t think we have the education to do it. We’re not trained in nutrition at all. And I think we’re not comfortable doing it. Nor do I think we necessarily - that should be our role” (MD 2). One doctor challenged this perceived barrier among PCPs, highlighting that it is their responsibility to educate themselves if necessary:

See you never ever get a -- an internist who would say ‘I know nothing about cardiology. I know nothing about what a cardiac cath is. But I’m also not so sure it’s so important.’ Yet ……- I could find some doctors [who] would say, ‘you know, I really don’t know that much about nutrition. I’m not really sure how important it is. And I don’t really know how to do it’ (MD 1).

Regardless of competency, some PCPs questioned whether or not they were the best members of the team to provide the counseling when dietitians had more time to dedicate to lifestyle counseling and had higher levels of expertise, especially when the patient had complex medical issues that only the PCPs could address.

“I don’t mind spending 5, 10 minutes -- even 20 minutes, the whole time, you know, talking about exercise, nutrition. However, if a patient comes in who has seven problems including congestive heart-related, etc., and I have to manage all that stuff, the truth is you probably don’t want me to spend that time [on nutrition]” (MD 1).

In contrast, few nurses (LPNs and RNs) cited lack of sufficient training and competency as reasons for not counseling patients. Personal interest and experience with weight management was a stronger factor influencing their perceived role. Those who had personal success losing weight and/or changing their lifestyle proudly described how they counseled patients about their weight and shared what they learned from their experiences. They provided specific tips on how to lose weight and felt that providing support to patients was important, “If they know you’re having a problem, [the patients] love it. They’re not alone. And I just say, well, this is what I do. You know I stopped doing this at night or I tried walking a little more. Walk an extra block. Get off the bus one block sooner” (RN 5). Unlike the nurses, none of the PCPs or MOVE! staff spoke of using personal experiences to encourage patients to improve their lifestyle and lose weight.

Weight management counseling roles were also informed by perceptions of proper team structure and variations in perceived responsibilities and capabilities. One RN who did not counsel patients about weight loss and the MOVE! program said that the doctor on her team preferred to do the counseling herself. Despite the fact that some of the LPNs counseled patients and provided specific diet and physical activity advice, PCPs did not always believe that LPNs had the skills to do so, “I mean I know my LPN is very nice but I don’t know how much she’d be able to counsel or help coordinate that” (MD 7).

#### Anticipated outcomes of weight management counseling and programs

Believing that VHA weight management programs and individual/team practices would lead to positive outcomes was a facilitator to engaging in providing weight management care. On a system level, outcome expectations included perceptions about the MOVE! program. At an individual level, these included feelings and perceptions about whether or not the Veterans could overcome barriers to weight loss and beliefs about the effectiveness of weight management counseling and goal setting.

#### System level factors

The amount of effort nurses and PCPs spent trying to get patients to go to the MOVE! program depended on their perception of its effectiveness. Those with a positive view of the MOVE! program were more likely to recommend it. While most of the PCPs and nurses tried to encourage patients to attend MOVE!, many questioned its effectiveness. “I think that providers don’t push for the MOVE! program as much as they could 'cause we don’t have any data that it’s successful. And it’s hard to recommend something if you don’t know if it’s gonna work. So that’s, personally, one of my barriers” (MD 3), and some could not think of any patients who had had success with the program.

Similarly, the MOVE! staff were not sure whether or not the MOVE! program was effective locally. While they had had some success stories, one wondered if the program was intense enough, “So, I guess that we’re revising it. Right now there’s an 8 week program and I personally don’t believe that 8 weeks is enough time to lose weight” (MOVE! Staff 1). While the MOVE! staff described certain portions of the group-based MOVE! program (such as food demonstrations) as very effective and popular with patients, they felt that they were able to have more of an impact when they met with clients individually. “…[we] all have success stories about patients that they’ve helped one-on-one. We don’t have a lot of success stories that have been told to me about people who only lost weight in the groups…. But, the research tells us the group learning on weight loss should be better” (MOVE! Staff 1). Both PACT and MOVE! staff described barriers to MOVE! participation including time, cost of travel, and the fact that MOVE! groups were only offered during work hours.

#### Individual/Team level factors

Perceptions about patient barriers influenced outcome expectations of both doctors and nurses. Many believed that it was difficult to motivate patients to make positive behavior changes, “Well, I find that the patient has got to want themselves to lose weight. I could want it for them but if they don’t want it for themselves it’s not going to work. It’s not going to work. And a lot of—some of the patients are lazy” (LPN 4). In contrast to patient motivation, many emphasized the role of psychosocial barriers that they felt decreased their likelihood of being able to impact patient behaviors. These barriers included homelessness, substance abuse, depression, and PTSD, “Many of our patients’ lives are very difficult…there’s so much mental health pathology and there’s so much financial stress, unemployment, sick family members…” (MD 6). Outside influences such as an unhealthy food environment and advertising were cited as additional challenges “The media…has beaten us. Which is ‘oh Doc, I’m doing so great. I’m drinking green tea every day.’ Really? He’s drinking Arizona Iced Green Tea and he’s an educated guy. And so we actually went over that and then I pulled up—Googled Arizona Green Tea-- and I showed him under their label and showed him the sugar contents and the carb contents and the calories, and he’s like, ‘ooh’” (MD 9).

Study participants generally had positive views about goal setting and felt that this was a useful counseling technique to promote specific behavioral changes “I think [having goals] gives the patient something to do when…they’re not at the [VA]. They leave thinking that okay I’ll try to do this so next time I see my nurse or my doctor I can say I reached my goal. I accomplished my goal” (LPN 4). Participants consistently spoke of the importance of working with the patients to set small, incremental behavior change goals and had received training on goal setting. They described, using similar language, their belief that eliciting goals from the patients themselves facilitated the goals setting process “I think that the most important thing is whatever the goal is, it should be primarily driven by the patient, not the doctor” (MOVE! Staff 3). However, they acknowledged that positive views about goal setting were not sufficient to guarantee that conversations about goals would occur given the time constraints and competing priorities.

#### VHA communication and information dissemination

Communication emerged as a theme that influenced weight management practices. At the system level, study participants cited several ways that the VHA communicated weight management priorities that guided practice. On an individual level, they noted inadequate communication about what happens to individual patients treated by dietitians and MOVE! staff as a barrier to care.

#### System level factors

EMR reminders were one way that the VHA communicated and helped to reinforce national VHA weight management-related priorities. This facilitated conversations with patients about the MOVE! program itself, but was a barrier to other forms of weight management counseling. Encouraging patients to attend the MOVE! program was perceived as a priority over weight management counseling by PACT teamlets because each VA site was graded on how many eligible patients attended MOVE! as a performance measure “So maybe because right now PACT requires so much work that we’re not focusing enough on obese or overweight, they leave that to the MOVE! clinic. Maybe if they decided to make that more of an issue or more of a goal for PACT, because that’s not one of the goals right now” (LPN 4). Thus, advising patients to go to MOVE! occurred more frequently than discussing weight loss, physical activity, and nutrition goals with patients during the primary care visit. Some appreciated this MOVE! reminder as a tangible task to address obesity “It’s just part of our care and there’s been, you know, this mandate to send them now to MOVE! through the clinical reminders …it’s gotten easier over time as they’ve simplified them …… So I mostly refer out” (MD 2). However, others felt that the reminder was an excuse to perform minimal counseling and believed it prevented patients from receiving high quality care “I don’t think having it as a reminder—good intentions--but I don’t think it serves what it should. As in, ‘oh, I just need to click off the reminder. Do you want to go? No? Okay.’ You know, and then you’re done with the reminder. So I don’t think it’s as helpful as other things can be” (MD 9).

System-level priorities were also communicated to staff by time allocation and counseling templates or scripts. For instance, LPNs and RNs felt encouraged by the VHA to provide dietary counseling for poorly controlled hypertensive patients during 30 min scheduled nursing visits. For these visits, they were given a template that prompted them to address dietary issues, which facilitated counseling for this subset of patients. However, they were not told to provide separate visits focused on weight management for the general population, and 3 RN care managers independently suggested that this would be helpful. RNs and LPNs felt that if the VHA provided more time for weight management counseling and follow up phone calls, then they would be able to more reliably provide patient education around weight management.

Another barrier was poor communication to PC staff about the MOVE! program. Despite the fact that the MOVE! staff reported trying to educate PACT teamlets about the MOVE! program through a variety of strategies, the majority of participants complained that they did not have a good understanding of the components of the MOVE! program. This generally came up in conversation after the interviewer asked “what aspects of the MOVE! program are most effective?” They felt that this lack of understanding negatively impacted their ability to describe the program to patients “I don’t think any of us have a good enough sense of what actually happens in the MOVE! Program… and I sort of want to drink some Kool-Aid about it to be motivated myself to get them in” (MD 7). Thus, several participants independently suggested that they be given time to sit in on the MOVE! groups in order to have a better understanding of the program. They also suggested that the MOVE! staff increase direct communication to patients about the MOVE! program through advertisements and by having patients who lost weight through MOVE! speak to potential new enrollees.

When making system-level suggestions for improving weight management care, some participants complained that they did not have a way to formally recommend and communicate potential changes to local and national leaders. They felt left out of the decision-making process because they were told to do things without having much of a voice about what happened “I noticed that, here in the VA, every time they want to do something, they right away just tell me, okay, you do this, you do that. Then they tell me it’s ready without even consulting with us” (RN 2). Thus, some participants were skeptical that changes that they proposed would be implemented.

#### Individual/Team level factors

Communication between the PACT teamlets and dietitians/MOVE! staff about the progress of individual patients was another barrier to weight management care. Almost every LPN, RN, and MD interviewed wanted more direct feedback about their patients’ progress in the MOVE! program and/or with individual dietitian visits so that they could more effectively follow up with the patient about dietitians’ recommendations. In comparison, the MOVE! staff perceived that they provided PACT team members with a lot of information through notes in the electronic medical record, but that these often were not read.

## Discussion

The VHA has been a leader in implementing weight management programs in the outpatient setting. By interviewing PACT teamlet and MOVE! staff, our study identified several system-level and individual-or team-level facilitators and barriers to obesity care that can be used to inform interventions to improve obesity care at the VA. While this study was done within the VA, we believe these results could be used as a starting point when exploring barriers and facilitators to implementing weight management services within primary care at non-VA sites that use the PCMH model of care.

The Consolidated Framework for Implementation Research (CFIR) is a useful model to understand how our results complement other studies of weight management services at the VA. Damschroder et al. used this model to explore which constructs of CFIR could distinguish low from high MOVE! implementation sites [26]. They assessed 4 out of 5 of the CFIR components (“Intervention Characteristics,” “Inner Setting,” “Outer Setting,” and “Implementation Process),” but did not examine the 5th component called “Individual Characteristics. ” This component refers to the characteristics of the individuals involved with the intervention and/or implementation process. These characteristics include knowledge and beliefs about the intervention, self-efficacy or beliefs about their own abilities/skills, their readiness to adopt the intervention, and their perceptions about the organization where they work [26]. Our study contributes to the literature by qualitatively examining individual characteristics and perceptions of MOVE! and PACT staff about the care of obese individuals. We discuss how our findings build upon prior studies and how they can inform improvements to the provision of weight management care.

On an individual level, the perception that counseling was an important part of one’s role facilitated weight management care. Themes related to perceptions of role and responsibility have emerged in other qualitative studies about health providers weight management practices as well [38, 39]. For instance, Chilsom et al. found that physicians in their study did not feel that behavior change talk was their responsibility. In our study, role perception was influenced by perceived competency (in PCPs) and personal experience with weight management (in nurses). Several studies have shown that doctors have poor training in obesity care [5, 39], do not feel competent [4], and are more likely to counsel patients if they have higher perceived competency [40]. A recent study of nurses also showed that perceived skills also positively influenced weight management practices of nurses [41]. In physicians, those who engage in physical activity are more likely to counsel patients [42, 43], and nurses and doctors who are of normal weight are more likely to provide weight management advice to patients [44]. Thus, increased obesity-specific training is important for primary care teams if they are expected to help reduce the obesity epidemic. Our study’s findings suggest that weight reduction training interventions might encourage healthcare teams to adopt healthier lifestyles, which may increase their likelihood of counseling patients.

While many nurses believed obesity-related counseling to be an important part of their role, our study suggests that nurses may be underutilized for obesity-related care. Some non-PCP staff, especially LPNs, did not counsel patients about weight because they did not think that this was permitted by their license and/or the PCP on their team did not perceive that they had the ability to do so effectively. Yet health coaches and peers, who have less training than LPNs, can successfully help patients to lose weight [45]. Further studies are needed to explore team dynamics with regard to obesity counseling and why these differences in role perception exist. The Interprofessional Education Collaborative recommends clear roles, and responsibilities facilitate high performing teams [46]; therefore, encouraging better division of labor among all members of the team could increase the likelihood that patients would receive counseling and reinforcement during a visit.

Since most of the participants had a positive view of goal setting and most had received specific training about SMART goals [37], integrating goal setting into counseling practices may improve team-based weight management care. LPNs and RNs could help the patient set initial weight management and lifestyle goals, and the providers could further endorse goals and address potential barriers to achieving them. This would potentially encourage behavior change, even for patients who decide not to attend an intensive weight management program like MOVE!. If time and staffing barriers prohibit this, promising technological solutions such as clinician support tools, online resources, and smart phone apps could potentially facilitate goal setting. Further studies are needed to determine how to best integrate goal setting into PACT work flow and how dietitians and PACT teamlets can work together to help patients set and achieve their goals.

On a system level, participants indicated that the VHA communicated weight management priorities through time and resource allocation. Time and competing demands were barriers to weight management counseling, and this has been shown to be true in other VA and non-VA settings [3, 29]. Staffing constraints with MOVE! and PACT implementation are known barriers to care [29, 47, 48], and we found this to be true in our study as well. Thus, strategies such as providing weight management appointments with staff may help PACT teamlets prioritize weight management. For example, nurses reported they were more likely to counsel patients during blood pressure visits when they had dedicated time for lifestyle counseling. If this is not possible, adding a member to the team such as a health coach to provide lifestyle counseling to patients, help set initial health goals, and facilitate communication with PACT teamlets is a reasonable alternative. If resources do not allow for additional team members to improve staffing, evidence suggests that these barriers may be ameliorated as PACT teamlet function improves overall [48]. Either way, our study supports eliciting recommendations from staff themselves and communicating to staff that weight management counseling is a VHA priority despite competing demands.

Technology could either help to bridge or complicate weight management practices in primary care. A recent systematic review showed that technological solutions can promote weight loss and lifestyle change in the primary care setting [49]. The VHA also recently released the publically available MOVE! Coach Mobile, which helps patients set goals and monitor their lifestyle behaviors from their smartphone. In contrast, while EMR reminders increase rates of BMI screening [50], our study suggests that VHA reminders may actually be a barrier to weight management counseling by PACT teamlets since there is a focus on sending patients to the MOVE! program rather than having the teams directly provide weight management care (in addition to referring to MOVE!). Requiring a high volume of clinical reminders as a way to measure performance may also overwhelm staff. A recent study found that there was a perception among staff that such performance measures do not truly measure patient-centered quality [51]. Thus, providing more in depth evaluation and feedback of counseling practices through observation or patient exit interviews may be warranted to assess actual practice, enhance competence through feedback, and improve workflows within the system.

Finally, despite the VHA focus on sending patients to MOVE!, the majority of PACT teamlet staff did not know what their patients experience during MOVE! sessions. This was due to suboptimal communication and may have also influenced KIs’ perceptions about the effectiveness of the MOVE! program. Encouraging PACT staff to participate in the MOVE! program either as a participant or as an observer may be one way to give them more information to encourage patients to go to the program. Further, we found that there was a need to improve communication between MOVE! and PACT staff about outcomes with individual patients. Damschroder et al. described that communication between MOVE! Coordinators and PCPs facilitated MOVE! referrals and was stronger at high implementation MOVE! sites than low implementation sites [27]. One way to improve communication may be to have MOVE! dietitians more closely embedded into PACT team meetings to discuss patient issues in person.

While this study was conducted at the VA, results and recommendations have implications in non-VA settings. Given that the PCMH model is being increasingly adopted in primary care, we need to understand how to leverage this model to facilitate weight management counseling outside of the VHA. When implementing weight management programs, institutions with PCMH models of care may anticipate similar system, team, and individual-level barriers (e.g. lack of time and competing demands) and may want to ensure that known facilitators are present (e.g. systematic training of staff ). Further, the VHA has infrastructure that facilitates delivery of weight management services (e.g. clinical reminders, the MOVE! program, online MOVE! resources), so this work may help other institutions determine the need for such infrastructure and how to best implement it. Patients are more likely to lose weight when they participate in intensive weight management programs. While other organizations may not have onsite programs like MOVE!, they similarly struggle with how to improve patient adherence to weight management programs in the community (e.g. YMCA Diabetes Prevention Program and Weight Watchers). Additionally, this research can inform other studies exploring barriers and facilitators to weight management care in other PCMH settings.

### Limitations

This was a single-center qualitative study, and findings may be unique to our urban US east coast location. However, our findings are supported by the literature, and we expect that this site faces many of the same challenges to PACT and MOVE! implementation and integration as other VA sites. Since interviews occurred at the hospital site, interviewees may have felt inhibited while in their workplace. To address this, we made sure the interviews occurred outside of their clinical time, and consent measures helped ensure confidentiality. Further, the PI of the study worked as a physician on a PACT team. While this ensured that the research questions were grounded in real-world practice, it may have introduced bias. Several steps were taken to minimize bias including having a second independent coder as well as an outside qualitative methods expert with a nursing background involved in all aspects of research question formulation and data analysis. Further, we confirmed results with the participants. While gender is known to be a strong predictor of weight management counseling [44], we could not explore gender differences in responses because the majority of our participants were female. Additionally, while it would have been interesting to explore team dynamics, given confidentiality, privacy, and professional concerns, we could not analyze data by team. Finally, we were only able to recruit one clerical assistant and this person’s views may not be representative of the role as a whole. Despite these limitations, the relevance and potential application of our findings provide a foundation for initial recommendations to improve delivery of care to overweight and obese Veterans in the PC setting.

## Conclusion

Our data suggest that there are a diversity of weight management-related attitudes and counseling practices among PACT teamlet and MOVE! staff influenced by role perception, program outcome expectations, and communication issues. These findings inform our suggestions for how to improve implementation of weight management care within PACT and increase participation in the MOVE! program. Further, they may help inform delivery of weight management services within PCMH models of care at other institutions. Future research is needed both to confirm our findings among VA staff at other locations and to develop and test interventions to improve delivery of care to overweight and obese patients within primary care.

The views expressed in this article are those of the authors and do not necessarily reflect the policies or views of the Department of Veterans Affairs.

## Abbreviations

LPN: Licensed practical nurse
NP: Nurse practitioner
PACT: Patient aligned care teams
PC: Primary care
PCP: Primary care provider
RD: Registered dietitian
RN: Registered nurse
VA: Veterans Affairs Medical Centers
VHA: Veterans Health Administration
